# Supplementary material for: The Haplotype-Based Analysis of Aegilops tauschii Introgression Into Hard Red Winter Wheat and Its Impact on Productivity Traits
Source: Front Plant Sci. 2021 Aug 17;12:716955. doi: 10.3389/fpls.2021.716955 (PMC8416154; doi:10.3389/fpls.2021.716955)
Supplement: Supplementary file 1 [file Data_Sheet_1.zip › Figure_S1.pptx]

## Slide 1
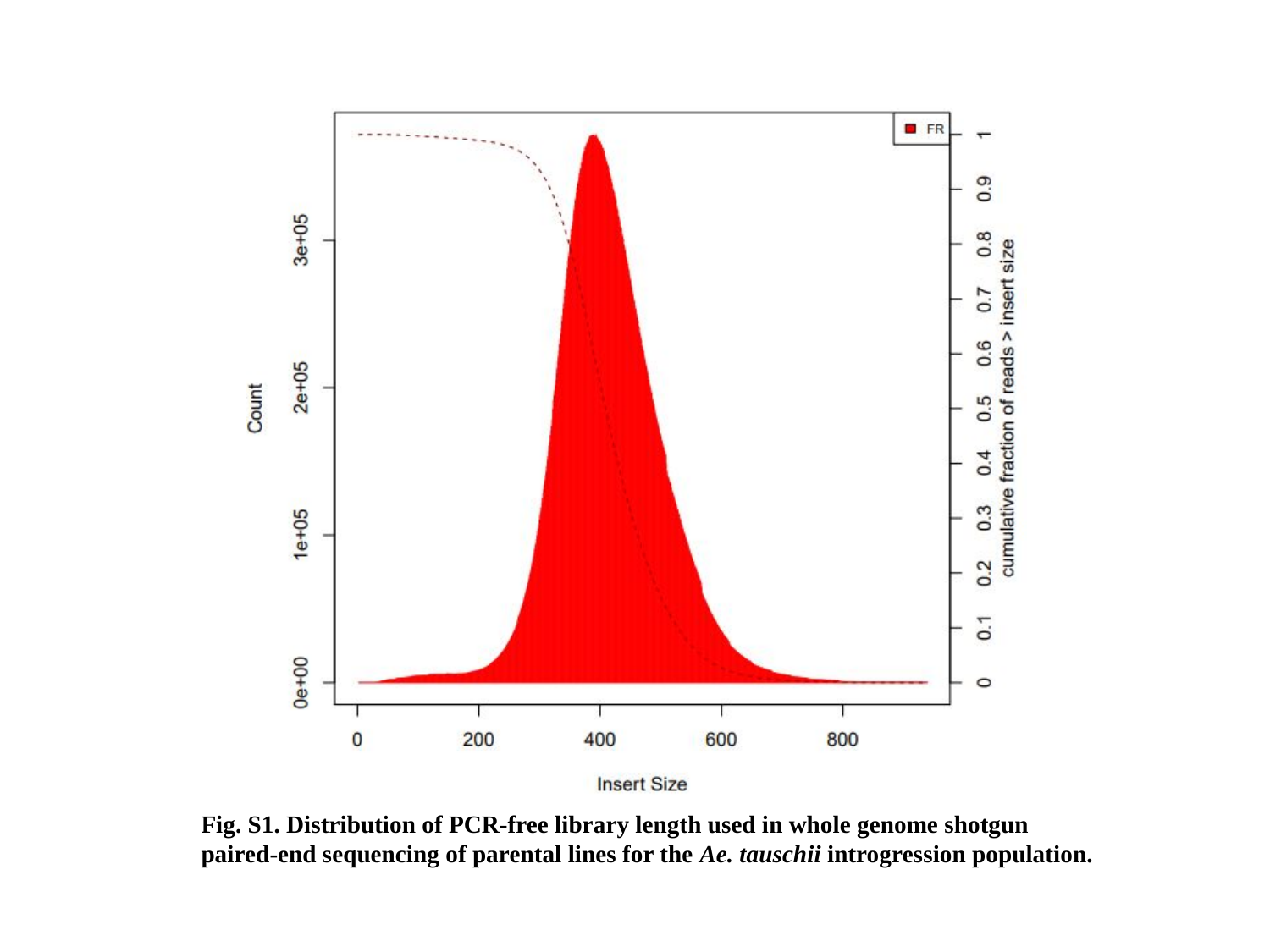

Fig. S1. Distribution of PCR-free library length used in whole genome shotgun paired-end sequencing of parental lines for the Ae. tauschii introgression population.
