# Supplementary material for: The Haplotype-Based Analysis of Aegilops tauschii Introgression Into Hard Red Winter Wheat and Its Impact on Productivity Traits
Source: Front Plant Sci. 2021 Aug 17;12:716955. doi: 10.3389/fpls.2021.716955 (PMC8416154; doi:10.3389/fpls.2021.716955)
Supplement: Supplementary file 1 [file Data_Sheet_1.zip › File_S5.pptx]

## Slide 1
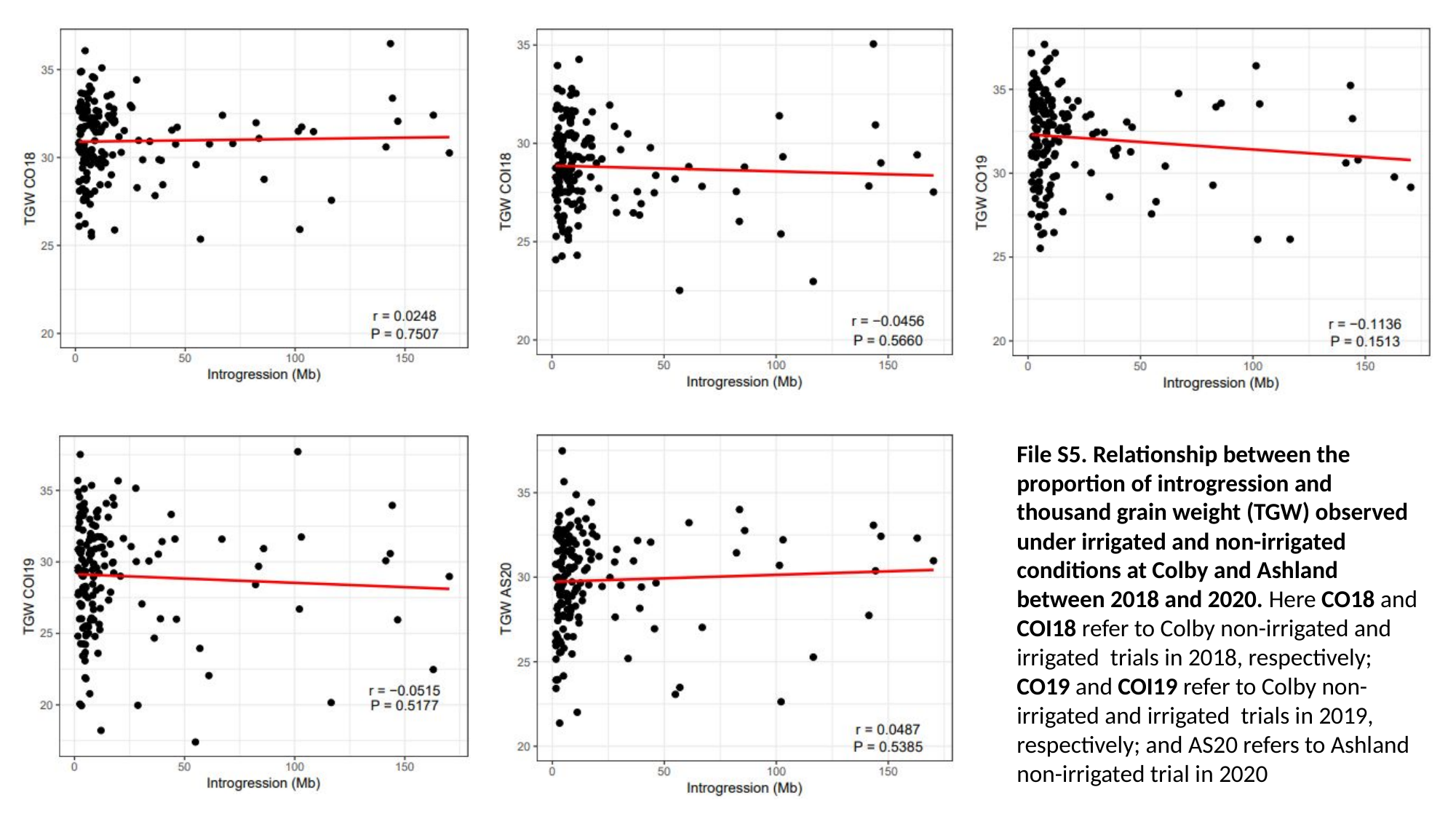

File S5. Relationship between the proportion of introgression and thousand grain weight (TGW) observed under irrigated and non-irrigated conditions at Colby and Ashland between 2018 and 2020. Here CO18 and COI18 refer to Colby non-irrigated and irrigated trials in 2018, respectively; CO19 and COI19 refer to Colby non-irrigated and irrigated trials in 2019, respectively; and AS20 refers to Ashland non-irrigated trial in 2020

## Slide 2
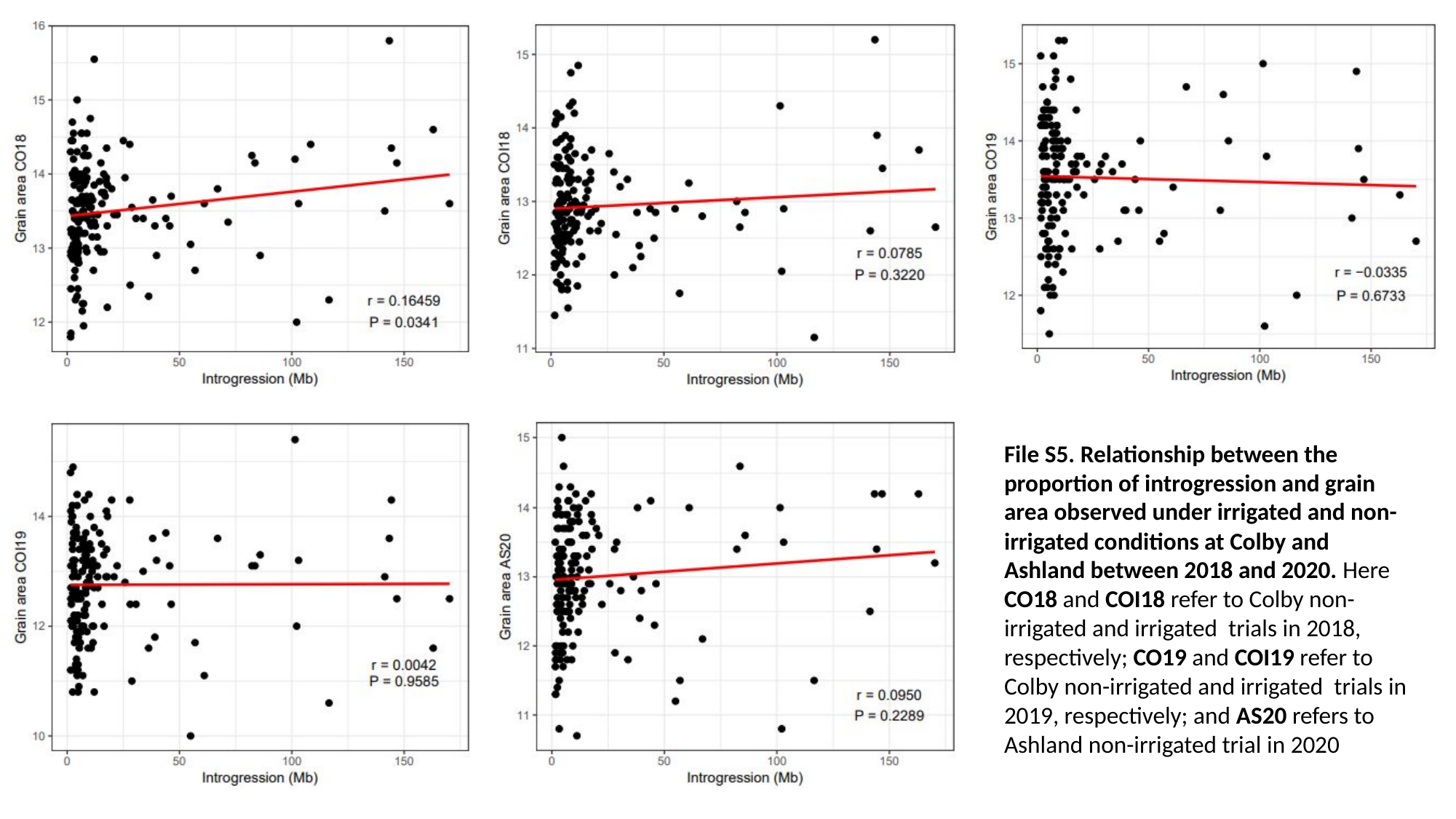

File S5. Relationship between the proportion of introgression and grain area observed under irrigated and non-irrigated conditions at Colby and Ashland between 2018 and 2020. Here CO18 and COI18 refer to Colby non-irrigated and irrigated trials in 2018, respectively; CO19 and COI19 refer to Colby non-irrigated and irrigated trials in 2019, respectively; and AS20 refers to Ashland non-irrigated trial in 2020

## Slide 3
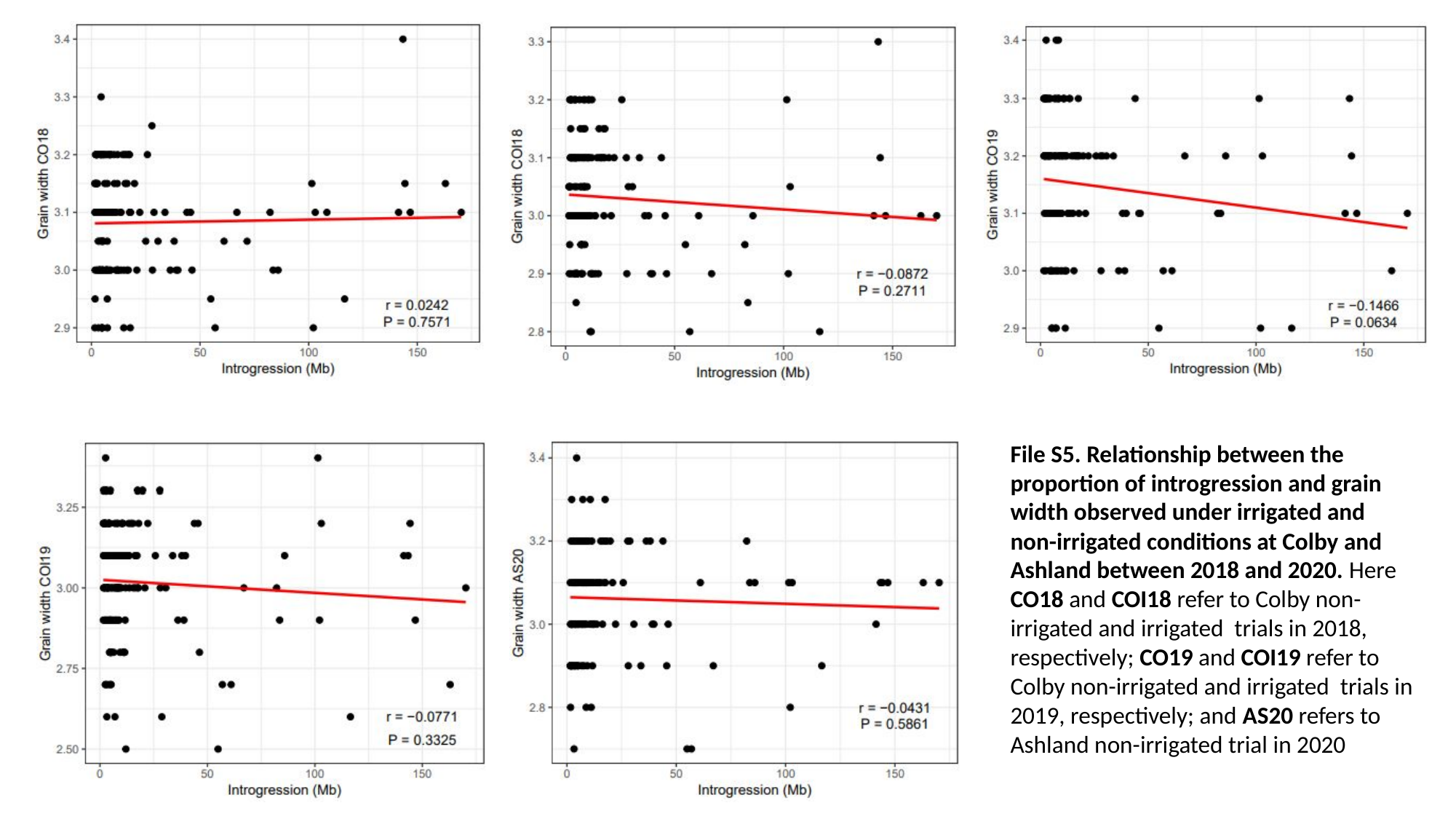

File S5. Relationship between the proportion of introgression and grain width observed under irrigated and non-irrigated conditions at Colby and Ashland between 2018 and 2020. Here CO18 and COI18 refer to Colby non-irrigated and irrigated trials in 2018, respectively; CO19 and COI19 refer to Colby non-irrigated and irrigated trials in 2019, respectively; and AS20 refers to Ashland non-irrigated trial in 2020

## Slide 4
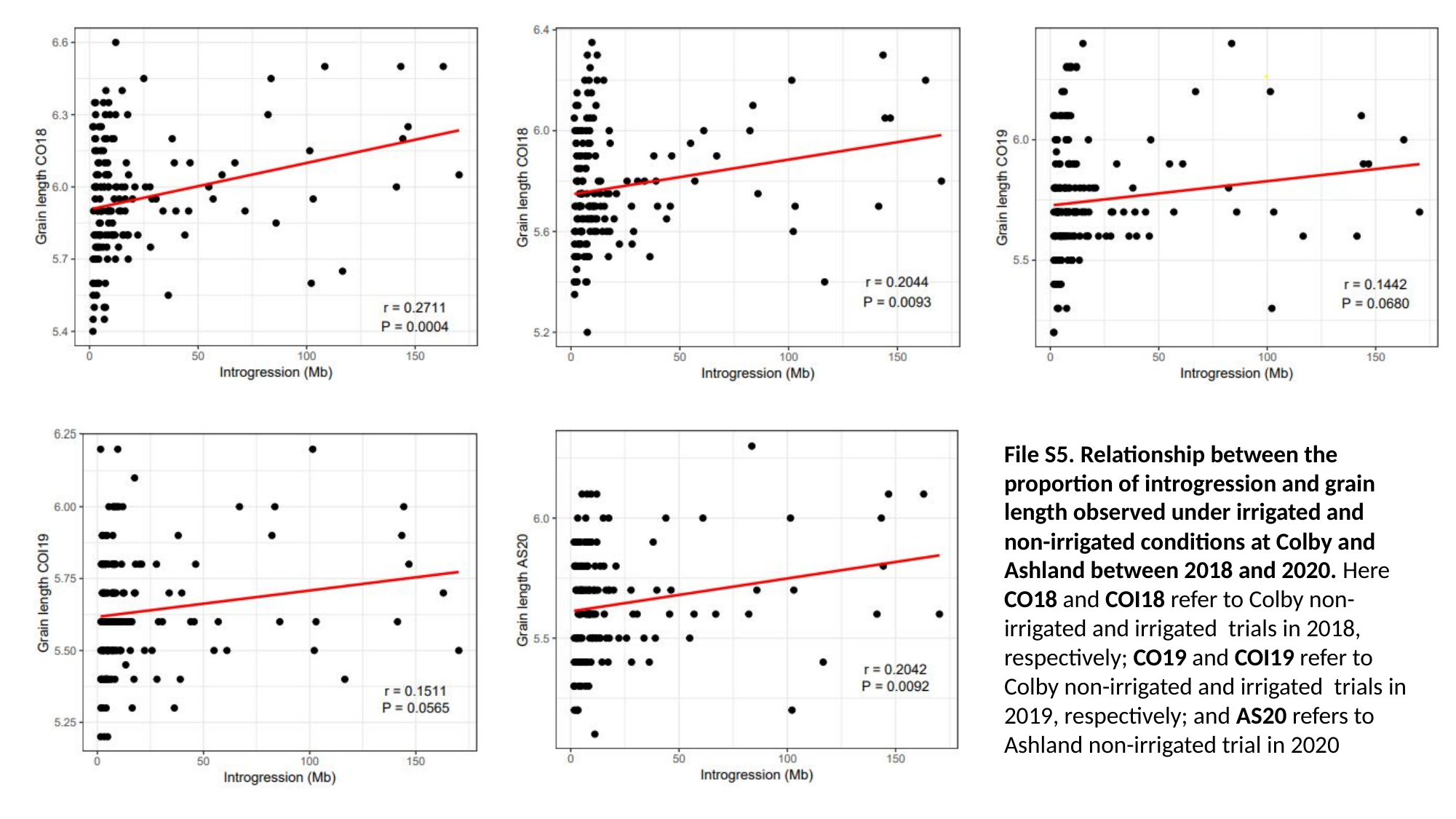

File S5. Relationship between the proportion of introgression and grain length observed under irrigated and non-irrigated conditions at Colby and Ashland between 2018 and 2020. Here CO18 and COI18 refer to Colby non-irrigated and irrigated trials in 2018, respectively; CO19 and COI19 refer to Colby non-irrigated and irrigated trials in 2019, respectively; and AS20 refers to Ashland non-irrigated trial in 2020

## Slide 5
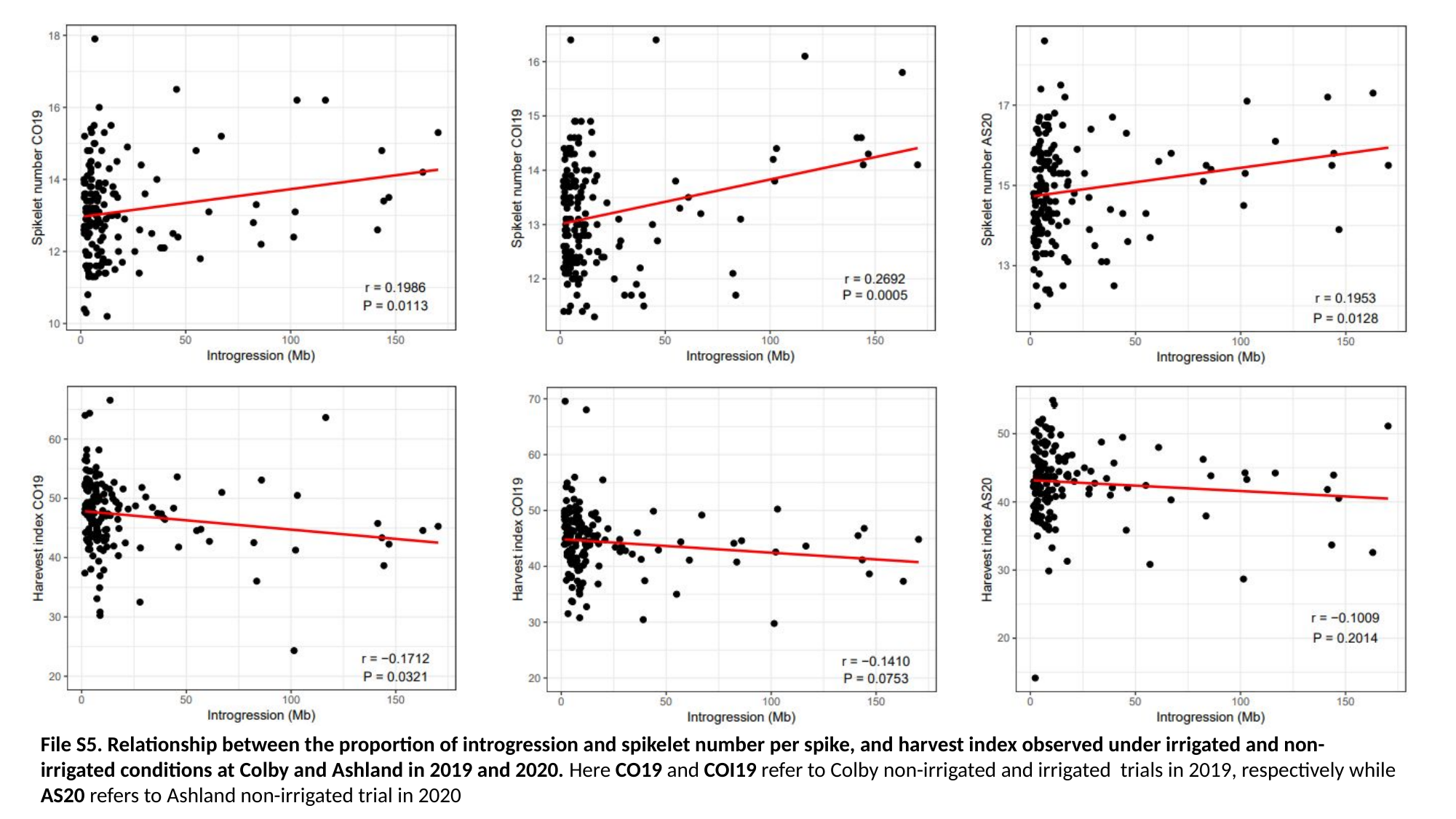

File S5. Relationship between the proportion of introgression and spikelet number per spike, and harvest index observed under irrigated and non-irrigated conditions at Colby and Ashland in 2019 and 2020. Here CO19 and COI19 refer to Colby non-irrigated and irrigated trials in 2019, respectively while AS20 refers to Ashland non-irrigated trial in 2020

## Slide 6
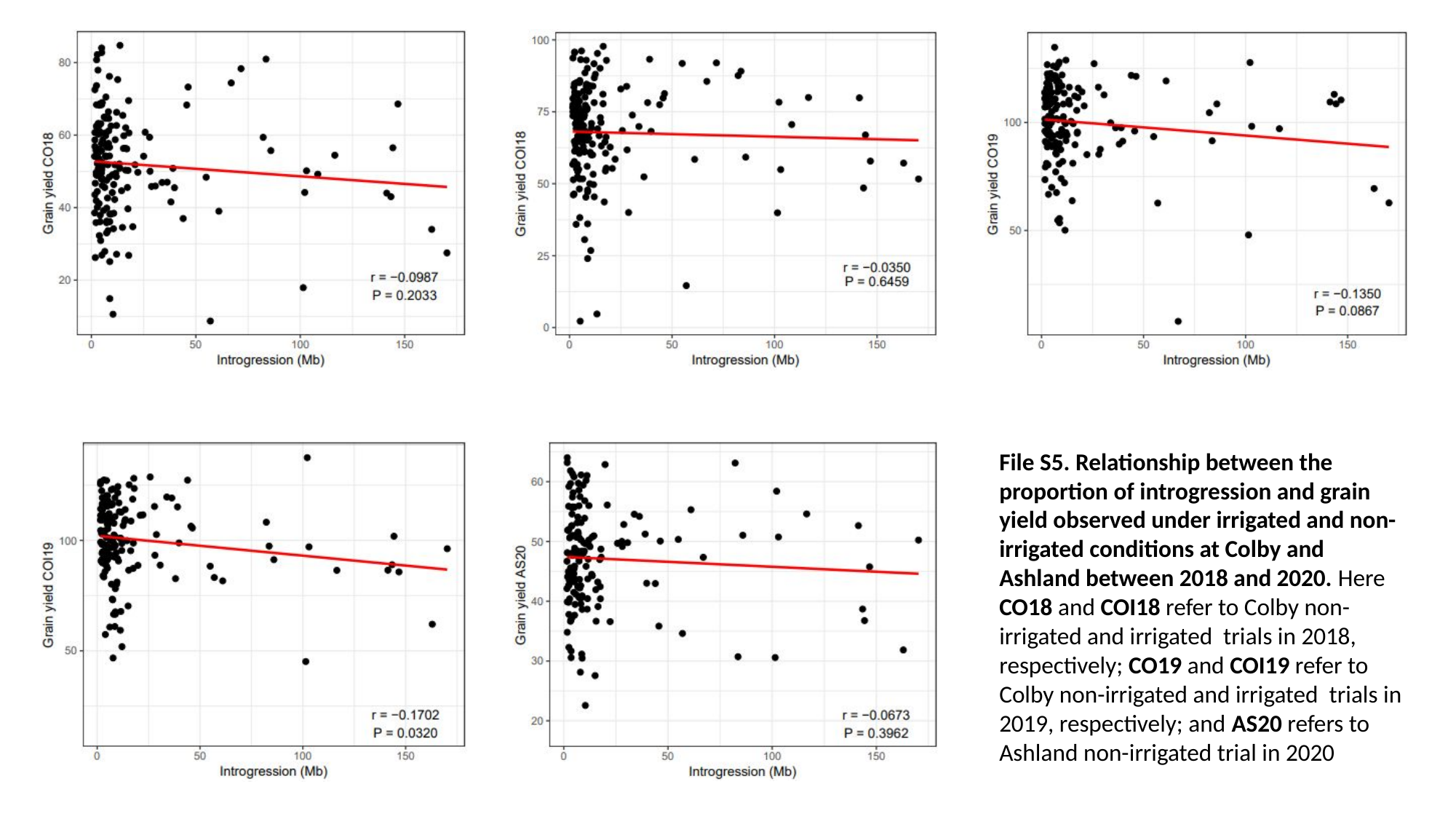

File S5. Relationship between the proportion of introgression and grain yield observed under irrigated and non-irrigated conditions at Colby and Ashland between 2018 and 2020. Here CO18 and COI18 refer to Colby non-irrigated and irrigated trials in 2018, respectively; CO19 and COI19 refer to Colby non-irrigated and irrigated trials in 2019, respectively; and AS20 refers to Ashland non-irrigated trial in 2020
